# Sodium currents in naïve mouse dorsal root ganglion neurons: No major differences between sexes

**DOI:** 10.1080/19336950.2023.2289256

**Published:** 2023-12-06

**Authors:** Mohammad-Reza Ghovanloo, Sidharth Tyagi, Peng Zhao, Philip R. Effraim, Sulayman D. Dib-Hajj, Stephen G. Waxman

**Affiliations:** a Department of Neurology, Yale University School of Medicine, New Haven, CT, USA; b Center for Neuroscience & Regeneration Research, Yale University, West Haven, CT, USA; c Neuro-Rehabilitation Research Center, Veterans Affairs Connecticut Healthcare System, West Haven, CT, USA; d Medical Scientist Training Program, Yale University School of Medicine, New Haven, CT, USA; e Department of Anesthesiology, Yale University School of Medicine, New Haven, CT, USA

**Keywords:** Dorsal root ganglion, voltage-gated sodium channel, excitability, patch-clamp, pharmacology, sex-dependence, action potential, TTX, neuron

## Abstract

Sexual dimorphism has been reported in multiple pre-clinical and clinical studies on pain. Previous investigations have suggested that in at least some states, rodent dorsal root ganglion (DRG) neurons display differential sex-dependent regulation and expression patterns of various proteins involved in the pain pathway. Our goal in this study was to determine whether sexual dimorphism in the biophysical properties of voltage-gated sodium (Nav) currents contributes to these observations in rodents. We recently developed a novel method that enables high-throughput, unbiased, and automated functional analysis of native rodent sensory neurons from naïve WT mice profiled simultaneously under uniform experimental conditions. In our previous study, we performed all experiments in neurons that were obtained from mixed populations of adult males or females, which were combined into single (combined male/female) data sets. Here, we have re-analyzed the same previously published data and segregated the cells based on sex. Although the number of cells in our previously published data sets were uneven for some comparisons, our results do not show sex-dependent differences in the biophysical properties of Nav currents in these native DRG neurons.

## Introduction

Chronic pain is the most prevalent health problem in the world [1–3]. Pain has two components, which include nociception, in which the neural encoding of actual or impending tissue damage is signaled, and the emotional and psychological component, in which the nociceptive signal is perceived in the central nervous system (CNS) as an unpleasant experience that is called pain [4]. An added layer of complexity in pain physiology is related to sexual dimorphism [3,5–7].

Dorsal root ganglion (DRG) neurons are among the most complex biological cell types, whose diversity and role as critical effector organs of the sensory pathway, including pain, is well-established [8–11]. DRG neurons express many different types of ion channel and receptor ensembles in various stoichiometries [8,9,12–16]. Among these proteins, the voltage-gated sodium (Nav) channel family are particularly interesting, as their role within the excitability pathway in DRG neurons is well-substantiated [10,14,17]. Previous studies have shown that the complement of Nav channels (Nav1.1/6–9) expressed in DRG neurons varies with cell size. Nav1.7 and Nav1.8 are expressed in neurons of different sizes, but the remaining subtypes that are sensitive to tetrodotoxin (TTX) are predominantly found in larger neurons, and those that are resistant to TTX are found in smaller cells [14,18,19].

The presence of sex-dependent differences in chronic pain has been long suggested by numerous large-scale studies that have investigated various types of pain, including musculoskeletal and arthritic pain, among many others [20,21]. Epidemiological studies suggest that women generally report more chronic pain than men [3]. Furthermore, preclinical studies suggest that sex-dependent differences in the hormonal regulation of nociceptors could contribute to variation in pain phenotypes among sexes [22–25]. Previous studies have shown that rodent DRG neurons display differential sex-dependent regulation and expression patterns of various proteins involved in the pain pathway. However, sex-dependent effects on biophysical properties of Nav channels within DRG neurons have not been previously investigated.

One approach to determining whether the biophysical properties of the Nav channels is different in DRG neurons from males versus females would be to assess sodium currents via a head-to-head and unbiased comparison of DRG neurons under identical conditions. This type of analysis has previously been challenging to achieve because of the low throughput nature of manual patch-clamp electrophysiology, and because of the presence of multiple subpopulations of sensory neurons [12,13].

We recently developed a new method using an automated high-throughput patch-clamp platform, where we functionally characterized the Nav channel properties of freshly isolated rodent DRG neurons [9]. The published results in our previous study combined data from naïve wild-type (WT) adult C57BL/6 (7–9 weeks old) male and female mice, analyzed in an unbiased manner but not segregated on the basis of sex. In the present study we sought to determine whether Nav channel biophysical properties in DRG neurons varied as a function of sex. In this study, we have re-analyzed our previously published data, segregated by sex, and report that this analysis did not reveal sex-dependent differences in the biophysical properties of the Nav currents in populations of freshly isolated DRG neurons from male versus female adult C57BL/6 mice. To our knowledge, this is the first head-to-head comparison of Nav channel biophysical properties in sensory neurons from male versus female animals.

## Materials and methods

### Re-analysis of previously published data

All data presented in the manuscript were previously published [9]. Because experiments were done in separate cohorts of naive male and female mice, in this present manuscript, we divided up the previously published data into sexes, and re-plotted and re-analyzed, the data, which was re-binned (based on distribution mean). Methodological information entailing how data were acquired and analyzed is below.

### Preparation of DRG neurons from adult mouse

DRGs (at least 24 DRGs from each mouse) were harvested and immediately put in ice-cold complete saline solution (CSS) (in mM: 137 NaCl, 5.3 KCl, 1 MgCl_2_, 25 sorbitol, 3 CaCl_2_, and 10 HEPES, adjusted to pH 7.2 with NaOH). After all the DRGs were harvested, DRGs were transferred to 37°C enzyme solution −0.5 U/mL Liberase TM (Roche) and 0.6 mM EDTA in CSS for a 20-min incubation at 37°C, followed by a 15-min incubation at 37°C in another enzyme solution − 0.5 U/mL Liberase TL (Roche), 0.6 mM EDTA, and 30 U/mL papain (Worthington Biochemical) in CSS. DRGs were then centrifuged and triturated in 0.5 mL of 1.5 mg/mL BSA (low endotoxin) and 1.5 mg/mL trypsin inhibitor (Sigma) in DRG media [DMEM/F12 (Invitrogen) with 100 U/ml penicillin, 0.1 mg/ml streptomycin (Invitrogen), 2 mM L-glutamine (Invitrogen), and 10% fetal bovine serum (Hyclone)]. After trituration, un-dissociated pieces were removed by filtering through a 70-μm mesh (Becton Dickinson). To remove small supporting cells and small pieces of dissociated axons and myelin, density gradients with 15% BSA were applied twice. Cells were pelleted and re-suspended with 1 ml DRG media, layered on top of 15% BSA solution and centrifuged at 250 g for 10 min at 4°C; pelleted cells were then re-suspended with DRG media and went through a second round of 15% BSA purification. The cell pellet was re-suspended with 1 ml of DMEM/F12 (4°C) to get single-cell suspension. Five µl of cell suspension was counter stained with five µl of trypan blue to check neuronal number, viability, and purity. Single-cell suspension [225 ± 75 K (mean ± SD) live neurons total] was diluted to 3 ml in DMEM/F12 (4°C) before delivering to the 384 well chip on the Qube-384 instrument (Sophion A/S, Copenhagen, Denmark). An aliquot of the cell suspension was plated on a 35-mm Tissue Culture-treated culture dish and imaged using a Nikon microscope (Eclipse TE2000-U Inverted Microscope).

### Automated patch-clamp

Automated patch-clamp recording was used for all experiments. Sodium currents were measured in the whole-cell configuration using a Qube-384 automated voltage-clamp system. Intracellular solution contained (in mM): 120 CsF (or KF for CC experiments), 10 NaCl, 2 MgCl_2_, 10 HEPES, adjusted to pH7.2 with CsOH. The extracellular recording solution contained (in mM): 145 NaCl, 3 KCl, 1 MgCl_2_, 1.5 CaCl_2_, 10 HEPES, adjusted to pH7.4 with NaOH. Liquid junction potentials calculated to be ~7 mV were not adjusted for. Currents were low pass filtered at 5 kHz and recorded at 25 kHz sampling frequency. Series resistance compensation was applied at 100% and leak subtraction enabled. The Qube-384 temperature controller was used to maintain recording for all experiments at 22 ± 2°C at the recording chamber. Appropriate filters for series resistance (<10 MOhm) and Nav current magnitude (more than baseline in the inward direction, 0 nA) were routinely applied to exclude poor quality recordings. Data analysis was performed using Analyzer (Sophion) and Prism (GraphPad Software Inc., La Jolla, CA, USA) software. More relevant information could be found in Supplemental Information.

### Activation protocols

To determine the voltage-dependence of activation, we measured the peak current amplitude at test pulse potentials ranging from −120 mV to +30 mV in increments of +5 mV for 500 ms. Channel conductance (G) was calculated from peak I_Na_:

(1) $$G_{Na}=I_{Na}/\left(V-E_{Na}\right)$$

where G_Na_ is conductance, I_Na_ is peak sodium current in response to the command potential V, and E_Na_ (measured on IV relationships) is the Nernst equilibrium potential. Calculated values for conductance were fit with the Boltzmann equation:

(2) $$G/G_{max}=1/\left(1+exp\left[V_{1/2}-V_{m}\right]/k\right)$$

where G/G_max_ is normalized conductance amplitude, V_m_ is the command potential, V_1/2_ is the midpoint voltage and k is the slope.

### Steady-state inactivation protocols

The voltage-dependence of fast-inactivation was measured by preconditioning the channels from −120 to +30 mV in increments of 5 mV for 500 ms, followed by a 10 ms test pulse during which the voltage was stepped to −20 mV. Normalized current amplitudes from the test pulse were fit as a function of voltage using the Boltzmann equation:

(3) $$I/I_{max}=1/\left(1+exp\left[V_{1/2}-V_{m}\right]/k\right)$$

where I_max_ is the maximum test pulse current amplitude.

### Double Boltzmann for both activation and inactivation

Double Boltzmann fit was using the following equations:

(4) $$B=exp\left(\left(x-x01\right)/k1\right)$$

(5) $$C=exp\left(\left(x-x02\right)/k2\right)$$

(6) $$Y=y0+A*\left(p/\left(1+B\right)+\left(1-p\right)/\left(1+C\right)\right)$$

Where y0 is offset, A is span, x01 and x02 are midpoints, k1 and k2 are slope factors, and p is fraction.

### Recovery from inactivation protocols

Recovery from inactivation was measured by holding the channels at −120 mV, followed by a depolarizing pulse to 0 mV, then the potential was returned to −120 mV for different time periods. This was followed by a depolarizing 10 ms test pulse to 0 mV to measure availability. Recovery from inactivation was measured after pre-pulse durations of 20 ms and 500 ms and fit with a bi-exponential function of the form:

(7) $$\begin{array}{rl} & SpanFast=\left(Y0-Plateau\right)*PercentFast\\ & \;\;\;\;\;\;\;\;\;\;\;\;\;\;\;\;\;\;\;\;\;\;\;\;\;\;\;\;\;*0.01 \end{array}$$

(8) $$\begin{array}{rl} & SpanSlow=\left(Y0-Plateau\right)*\left(100-PercentFast\right)\\ & \;\;\;\;\;\;\;\;\;\;\;\;\;\;\;\;\;\;\;\;\;\;\;\;\;\;\;\;\;\;*0.01 \end{array}$$

(9) $$Y\;=\;Plateau\;+\;SpanFast\;*\;exp\left(-KFast\;*\;t\right)\;+\;SpanSlow\;*\;exp\left(-KSlow\;*\;t\right)$$

where t is time in seconds, Y0 is the Y intercept at *t* = 0, KFast and KSlow are rate constants in units the reciprocal of t, PercentFast the fraction of the Y signal attributed to the fast-decaying component of the fit.

### Quantification and statistical analysis

Normalization was performed in order to control the variations in sodium channel expression and inward current amplitude and in order to be able to fit the recorded data with a Boltzmann function (for voltage-dependences) or a biexponential function (for time courses of inactivation). The Sophion Qube is an automated electrophysiology instrument that is blinded to cell selections and experimentation, and selection is performed in a randomized manner. All subsequent data filtering and analysis is performed in a non-biased manner, in which automated filters are applied to the entire dataset from a given Qube run. Fitting and graphing were done using Prism 9 software (Graphpad Software Inc., San Diego, CA), unless otherwise noted. We performed the following two-stage statistical procedure for all group comparisons. First, we performed testing for normality using the Shapiro–Wilks test. If the underlying data distribution was normal, we performed parametric statistical testing – One-way analysis of variance (ANOVA) with Bonferroni correction: when each condition was being compared other conditions; or t-test: when overall 2 conditions were being compared. If the distribution of data was non-normal, we compared groups using non-parametric testing using the Kruskal Willis test with Bonferroni correction (when there were more than 2 groups) or the Mann–Whitney U-test (for 2 groups) [26]. A level of significance α = 0.05was used with p-values less than 0.05 being considered to be statistically significant. All values are reported as means ± standard error of means (SEM) or errors in fit, when appropriate, for n recordings/samples. Values are presented as mean ± SEM with probability levels less than 0.05 considered significant. The declared group size is the number of independent values, and that statistical analysis was done using these independent values. The numbers of cells in each of the experiments is provided in Table 1.

**Table 1.** Number of cells in each experiment/condition for male and females.

Protocol	Male	Female
Activation	11	13
500 ms recovery from inactivation small	22	26
500 ms recovery from inactivation large	11	20
20 ms recovery from inactivation small	25	24
20 ms recovery from inactivation large	4	8
500 ms recovery from inactivation small	24	26
500 ms recovery from inactivation large	3	10
TTX Inhibition	17	16
Spikes	19	10

## Results

### Nav channel activation in rodent DRG neurons is not sex-dependent

Each of our experimental preparations included pooled neurons from 3 to 4 adult male or female mice. Upon the completion of cell preparation, the freshly isolated pooled neurons were placed on our automated patch-clamp platform. The experimenter was blinded to the sex of the mice that were used in each run until the completion of data analysis, at which point the data were decoded. All electrophysiological experiments, including cell selection, and subsequent analyses were performed in an unbiased and automated manner using the novel approach that we recently developed (see: Ghovanloo et al. 2023) [9].

**Figure 1.**
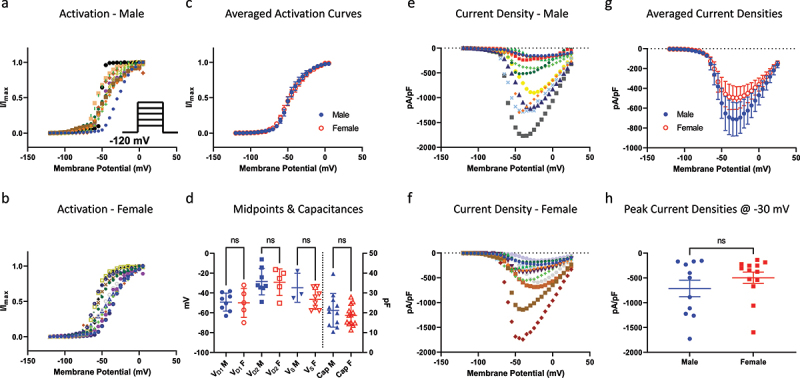
Activation of Nav channels in freshly isolated DRG neurons. a-b) all the GV data from each neuron. Each relationship displays normalized conductance as a function of membrane potential. c) averaged GV curves. d) distribution of individual midpoints on the left axis, and capacitances on the right axis. e-f) Current densities for each sex. g) averaged current densities. h) peak current densities measured at −30 mV.

The first biophysical property we investigated was activation. To do this, we used a standard step-pulse to elicit peak conductance at membrane potentials between −120 and +30 mV (Figure 1). We used capacitance of cells as a proxy for size [27]. As Nav channels have different voltage-dependence and kinetic properties, and because DRG neurons of different sizes are known to express different Nav channel isoforms, we fit the normalized conductance-voltage (GV) relationship of every neuron with both a single and a double Boltzmann function. Depending on whether the datum was better fit with either equation, the midpoints (V_D1_ and V_D2_, for double Boltzmann, and V_s_ for single Boltzmann) were binned together [9]. In Figure 1a,b, we show the individual GV relationships for cells that came from either male or female mice. We also show the averaged relationships for both sexes, which indicate an overlap of mean curves (Figure 1c). In Figure 1d, we show that both the capacitances, and hence neuronal sizes between the population of cells between sexes, and all activation voltage-dependent midpoints are not different from one another. These data suggest that in our cohort of neurons from males and females, the tested populations were of similar diameters, and that within these diameters, the activating properties of Nav channels were not significantly different (*p* > 0.05).

Figure 1e,h display the current densities associated with this cohort of cells. This was measured as peak sodium current divided by membrane capacitance (pA/pF), and plotted as a function of membrane potential (Figure 1e,f). Consistent with the activation data, the resulting peak current densities were not significantly different between sexes (*p* > 0.05) (Figure 1g,h).

### No significant differences in the voltage-dependence of steady-state inactivation (SSI) between sexes

**Figure 2.**
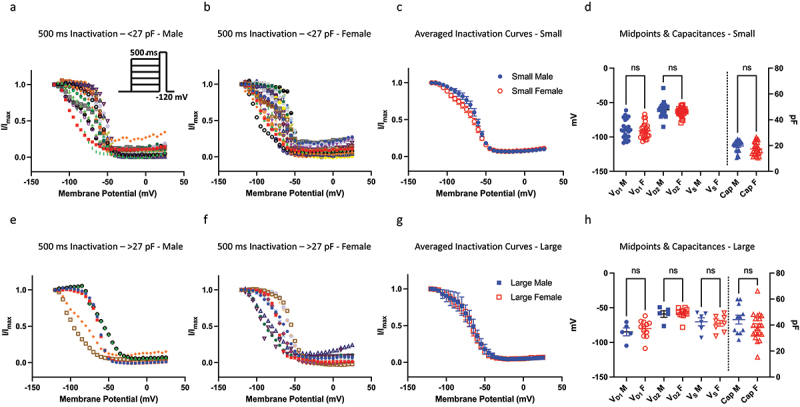
Steady-state inactivation of Nav channels. a-b) the current-voltage (IV) relationship for neurons within the <27 pF capacitive bin. The neurons within this bin have a clear bi-phasic distribution, and most of the Nav channels in these cells do not fully inactivate, i.e. there is a residual persistent current, as indicated but the low ends of the IV curves. c) averaged curves. d) distribution of individual midpoints on the left axis, and capacitances on the right axis. e-h) same info as above but for >27 pF.

Next, we assessed the voltage-dependence of SSI of DRG neurons obtained from male and female mice from a 500-ms pre-pulse duration (Figure 2). A 500-ms duration is considered to trigger an overall macroscopic intermediate inactivation, in which some channels undergo fast, and others undergo slower inactivated states [9,28,29]. In these experiments, we recorded from neurons ranging from about 10 to 75 pF, with the mean capacitance for both sexes being around 27 pF. Based on this range of capacitances, we divided up the neurons into two size bins, in which <27 pF were considered small and >27 pF were considered large. Each of the two sizes were further divided by sex. We applied the same single vs. double Boltzmann analysis to every datum, which expectedly suggested that overall, most cells are better described with double Boltzmann equation. Within the small bin, where a greater diversity of Nav channel expression (i.e. TTX-R/TTX-S ratios) is expected, every cell was better fit with a double Boltzmann. We found that there were no significant differences in the mean neuronal capacitance distributions between males and females, and that the resulting voltage-dependent midpoints were also not significantly different (*p* > 0.05). These results indicate that DRG neurons have similar steady-state inactivating properties between males and females.

### Sex-dependent differences in the kinetics of recovery from inactivation

An important attribute of Nav channels is their kinetics of recovery from various types of inactivation. In a simplified model, Nav channels can occupy three distinctive states, which include resting, activated, and inactivated, which can be further subdivided into fast and slow [30,31]. Whereas the molecular underpinnings for both types of inactivation are fundamentally different, from an electrophysiological perspective, they both depend on the magnitude and time-course of a depolarizing pulse.

**Figure 3.**
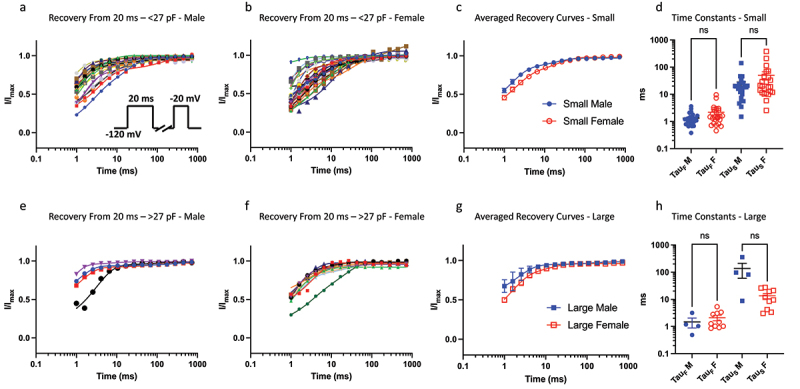
Recovery from 20 ms inactivation. a-b) data divided up using the same bins as above for 20 ms c) displays averaged data. d) distributions of time constants. e-h) same info but for larger sized cells. The sample size in the larger bin was small for males, which may obscure statistical comparison.

To measure recovery from fast inactivation, we held channels at −120 mV to ensure maximal Nav channel availability, then we pulsed the channels to −20 mV for 20 ms, which was followed by hyperpolarizing pulse back to −120 mV at increasing time intervals to allow recovery from inactivation. Finally, we pulsed the cells back to −20 mV as a test-pulse to measure amount of recovered Nav current (Figure 3). We previously found that larger neurons have faster recovery kinetics from fast inactivation from 20 ms, as indicated by the τ_Slow_ component (but not τ_Fast_) of their recovery [9]. Here, we compared these kinetic properties between males and females, and found that there are no significant differences in the τ_Slow_ between males and females (*p* > 0.05) in either size bins (Figure 3a–h).

**Figure 4.**
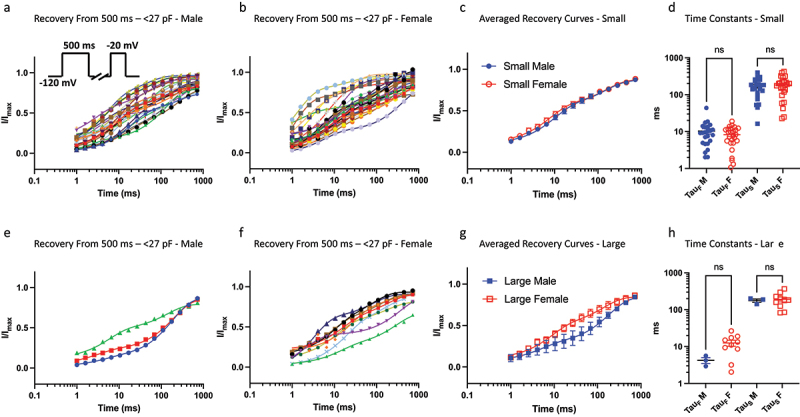
Recovery from 500 ms inactivation. a-b) data divided up using the same bins as above for 500 ms c) displays averaged data. d) distributions of time constants. e-h) same info but for larger sized cells. The sample size in the larger bin was small for males, which may obscure statistical comparison.

In our previous study we showed that because of the inherent variability in the gating properties of Nav channels, a 500-ms (intermediate macroscopic inactivation) pulse could cause the various Nav channel populations to become inseparable, as indicated by kinetic time constants [9]. Here, we divided up the cells between sexes to determine if within the size bins, the recovery kinetics from intermediate inactivation would be different. We did not observe a significant difference between the sexes in recovery from intermediate inactivation (*p* > 0.05) (Figure 4a–h).

### No significant differences in TTX-mediated block of Nav currents among sexes

**Figure 5.**
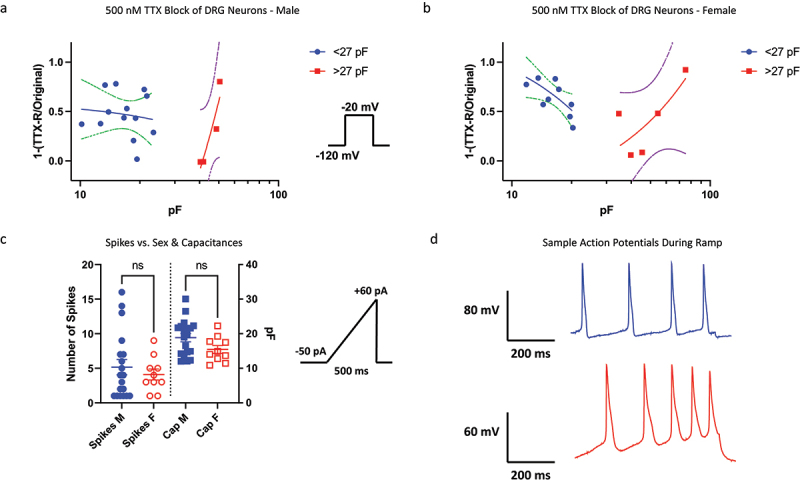
Pharmacological block by 500 nM TTX; excitability using current-clamp. a-b) the fraction of Nav current inhibited by TTX vs. capacitance. In cells with capacitances from ~20–40 pF for both sexes, there is a stronger presence of TTX-R (likely Nav1.8/9). The data were fitted with a simple linear regression equation to display the trend of TTX-sensitivity of cells as a function of capacitance. The dotted lines display the 95% confidence intervals. c-d) number of AP spikes that were elicited using a standardized ramp current-clamp protocol. Sample traces for males and females.

Nav channels in DRG neurons have differential sensitivities to TTX [32]. We previously showed that within our cohort of neurons, the smallest cells display variability in response to 500 nM TTX, and with the increase of cell size, the neurons show a decrease response to TTX (likely due to Nav1.8), with the largest end of the spectrum showing an increased TTX response (likely due to Nav1.6) [9]. Here, we broke up the data into male vs. female, and found that the overall trends between male and female cells were similar (Figure 5a,b). Therefore, consistent with the previous results, there do not seem to be major differences in the TTX-sensitivity of Nav currents between male and female neurons.

### No differences in cellular excitability between sexes

Finally, we sought to determine if there are differences in the excitability of DRG neurons between sexes. We elicited action potential firing using a standard ramp current protocol in which −50 pA of current was applied the cells, which was then ramped up to +60 pA over the course of 500 ms (Figure 5c,d). We did not observe significant differences between males and females in the capacitances nor the number of spikes that were generated (*p* > 0.05).

## Discussion

There are numerous reports of sexual dimorphism in various pain models [3]. These sex-dependent differences could result in reduction of the translatability of findings from pre-clinical rodent models to larger scale clinical studies. This becomes more problematic as a similar sexual dimorphism exists in human patients. As most preclinical studies describe sex-dependent difference in injured animals [3,5–7], our goal in this study was to determine whether there are significant sex-dependent differences in the characteristics of Nav current in male and female WT (uninjured) mouse DRG neurons, at baseline. Given the well-established role of Nav channels within the sensory pain pathway, we focused on these channels. To achieve this objective, we reanalyzed data obtained by a new method that permits recordings from freshly isolated, diverse neurons assessed simultaneously (head-to-head) under the same conditions [9].

DRG neurons are responsible for the detection of many sensory inputs [33]. The membranes of these complex cells are home to a variety of different ion channel and receptor proteins, which are present at different ratios and ensembles in a given neuron. These proteins and other downstream signaling pathways work in concert to produce complex signals. A change to any of the main components of the electrogenisome within these neurons could impact their functionality [10,16,33–35].

Our results in this study did not reveal major differences between males and females in the Nav currents within populations of DRG neurons of naive C57/BL6 mice. This is particularly important as previous studies suggest that various phenomena including injury-induced hormonal regulation of expression patterns of some vital ion channels within the sensory pathway (e.g. various TRP channels) sex-dependently [22,23]. It is conceivable that other ion channels and receptors could display sexual dimorphism even in naive baseline states. An important limitation in this study of previously published datasets is that the numbers of cells in some comparisons were uneven, which could have affected some of the noted conclusions. Nevertheless, given that we found no significant differences in the action potential firing of populations of neurons between sexes, we speculate that there may be no major differences in the biophysical properties of any of the vital components of the electrogenisome; this idea should be tested in future studies.

As noted in the Results section, Nav channels can exist in three fundamental states. Here, we found that, as studied on a neuronal population level, there are some but no major sex-dependent differences in these states. This has important implications for drug discovery. Given the vital role of Nav channels in pain, inhibition of these channels using different classes of compounds has been heavily pursued by academic laboratories and the pharmaceutical industry [28,36–38]. As such, research and development efforts often involve testing candidate compounds on primary sensory (e.g. DRG) neurons. We found that activation, steady-state inactivation, recovery from inactivation, as well as response to TTX were comparable between sexes. It is possible that the effects in large capacitance neurons could be different, but the number of neurons in our sample in this size class (>27 pF) is too small to allow us to make formal conclusions. This suggests that *in vitro* investigations of Nav-targeting compounds may be performed in small size WT primary cells from either sex.

In conclusion, we report that Nav currents within WT uninjured populations of DRG neurons from adult male and female mice display comparable biophysical properties. Our findings may have important implications for *in vitro* pharmacological studies.

## Availability of materials and data

Previously published data and material are available online, and/or will be available from corresponding author.

## References

[1] Relieving pain in America: a blueprint for transforming prevention, care, education, and research. National Academies Press; 2011 [cited 2021 Sep 5]. Available from: https://pubmed.ncbi.nlm.nih.gov/22553896/22553896

[2] James SL, Abate D, Abate KH, et al. Global, regional, and national incidence, prevalence, and years lived with disability for 354 diseases and injuries for 195 countries and territories, 1990–2017: a systematic analysis for the global burden of disease study 2017. Lancet 2018 [cited 2021 Sep 5];392:1789–11. Available from: http://www.thelancet.com/article/S0140673618322797/fulltext 30496104 10.1016/S0140-6736(18)32279-7 PMC6227754

[3] Mogil JS. Qualitative sex differences in pain processing: emerging evidence of a biased literature. Nat Rev Neurosci. 2020 [[cited 2021 Aug 14]];21:353. doi: 10.1038/s41583-020-0310-6. 32440016

[4] Mischkowski D, Palacios-Barrios EE, Banker L, et al. Pain or nociception? Subjective experience mediates the effects of acute noxious heat on autonomic responses. Pain . 2018 [cited 2023 Apr 16];159:699. doi: 10.1097/j.pain.0000000000001132. 29251663 PMC5901905

[5] Paige C, Plasencia-Fernandez I, Kume M, et al. A Female-Specific Role for Calcitonin Gene-Related Peptide (CGRP) in Rodent Pain Models. J Neurosci 2022 [cited 2023 Apr 16];42:1930–1944. Available from: https://pubmed.ncbi.nlm.nih.gov/35058371/35058371 10.1523/JNEUROSCI.1137-21.2022 PMC8916765

[6] Dedek A, Xu J, Lorenzo LÉ, et al. Sexual dimorphism in a neuronal mechanism of spinal hyperexcitability across rodent and human models of pathological pain. Brain. 2022 [cited 2023 Apr 16];145(3):1124–1138. Available from: https://pubmed.ncbi.nlm.nih.gov/35323848/35323848 10.1093/brain/awab408 PMC9050559

[7] Hadschieff V, Drdla-Schutting R, Springer DN, et al. Fundamental sex differences in morphine withdrawal-induced neuronal plasticity. Pain. 2020 [cited 2023 Apr 16];161(9):2022–2034. Available from: https://pubmed.ncbi.nlm.nih.gov/32345917/32345917 10.1097/j.pain.0000000000001901

[8] Dib-Hajj SD, Cummins TR, Black JA, et al. Sodium channels in normal and pathological pain. Annu Rev Neurosci. 2010 [cited 2018 Oct 3];33(1):325–347. Available from: http://www.ncbi.nlm.nih.gov/pubmed/20367448 20367448 10.1146/annurev-neuro-060909-153234

[9] Ghovanloo MR, Tyagi S, Zhao P, et al. High-throughput combined voltage-clamp/current-clamp analysis of freshly isolated neurons. Cell Rep Met. 2023 [cited 2023 Jan 12];3(1):100385. Available from: http://www.cell.com/article/S2667237522002909/fulltext 10.1016/j.crmeth.2022.100385 PMC9939380 36814833

[10] Dib-Hajj SD, Yang Y, Black JA, et al. The Na v 1.7 sodium channel: From molecule to man [Internet]. Nat Rev Neurosci. 2013 [[cited 2021 Oct 21]];14:49–62. Available from: https://pubmed.ncbi.nlm.nih.gov/23232607/23232607 10.1038/nrn3404

[11] Dib-Hajj SD, Waxman SG. Sodium channels in human pain disorders: genetics and pharmacogenomics [Internet]. Annu Rev Neurosci. 2019 [[cited 2021 Oct 19]];42:87–106. Available from: https://pubmed.ncbi.nlm.nih.gov/30702961/30702961 10.1146/annurev-neuro-070918-050144

[12] Middleton SJ, Barry AM, Comini M, et al. Studying human nociceptors: from fundamentals to clinic. Brain. 2021 [cited 2023 Oct 22];144(5):1312–1335. doi: 10.1093/brain/awab048 34128530 PMC8219361

[13] Zheng Y, Liu P, Bai L, et al. Deep sequencing of somatosensory neurons reveals molecular determinants of intrinsic physiological properties. Neuron [Internet]. 2019 [cited 2023 Oct 22];103(4):598–616.e7. Available from: https://pubmed.ncbi.nlm.nih.gov/31248728/31248728 10.1016/j.neuron.2019.05.039 PMC6706313

[14] Bennett DL, Clark XAJ, Huang J, et al. The role of voltage-gated sodium channels in pain signaling. Physiol Rev . 2019 [cited 2022 Jul 6];99:1079–1151. doi: 10.1152/physrev.00052.2017. 30672368

[15] Zamponi GW. Targeting voltage-gated calcium channels in neurological and psychiatric diseases. Nat Rev Drug Discov. 2015 [[cited 2023 Oct 22]];15:19–34. doi: 10.1038/nrd.2015.5 26542451

[16] Patapoutian A, Tate S, Woolf CJ. Transient receptor potential channels: targeting pain at the source. Nat Rev Drug Discov. 2009 [[cited 2023 Oct 22]];8:55–68. Available from: https://www.nature.com/articles/nrd2757 19116627 10.1038/nrd2757 PMC2755576

[17] Rush AM, Cummins TR, Waxman SG. Multiple sodium channels and their roles in electrogenesis within dorsal root ganglion neurons. J Physiol. 2007 [[cited 2018 Oct 3]];579:1–14. Available from: http://www.ncbi.nlm.nih.gov/pubmed/17158175 17158175 10.1113/jphysiol.2006.121483 PMC2075388

[18] Ramachandra R, McGrew SY, Baxter JC, et al. NaV1.8 channels are expressed in large, as well as small, diameter sensory afferent neurons. Channels (Austin). 2013 [cited 2022 Jul 6];7(1):34–37. Available from: https://pubmed.ncbi.nlm.nih.gov/23064159/23064159 10.4161/chan.22445 PMC3589279

[19] Shields SD, Ahn HS, Yang Y, et al. Nav1.8 expression is not restricted to nociceptors in mouse peripheral nervous system. Pain. 2012 [[cited 2022 Aug 8]];153:2017–2030. Available from: https://pubmed.ncbi.nlm.nih.gov/22703890/22703890 10.1016/j.pain.2012.04.022

[20] Fillingim RB, King CD, Ribeiro-Dasilva MC, et al. Gender, and pain: a review of recent clinical and experimental findings. J Pain [Internet]. 2009 [[cited 2023 Apr 16]];10:447–485. Available from: https://pubmed.ncbi.nlm.nih.gov/19411059/19411059 10.1016/j.jpain.2008.12.001 PMC2677686

[21] Mogil JS. Sex differences in pain and pain inhibition: multiple explanations of a controversial phenomenon. Nat Rev Neurosci. 2012 [cited 2023 Apr 16];13(12):859–866. Available from: https://pubmed.ncbi.nlm.nih.gov/23165262/23165262 10.1038/nrn3360

[22] Vandewauw I, Owsianik G, Voets T. Systematic and quantitative mRNA expression analysis of TRP channel genes at the single trigeminal and dorsal root ganglion level in mouse. BMC Neurosci. 2013 [[cited 2021 Aug 16]];14. doi: 10.1186/1471-2202-14-21 PMC3576292 23410158

[23] Patil MJ, Ruparel SB, Henry MA, et al. Prolactin regulates TRPV1, TRPA1, and TRPM8 in sensory neurons in a sex-dependent manner: contribution of prolactin receptor to inflammatory pain. Am J Physiol Endocrinol Metab . 2013 [cited 2021 Aug 16];305:E1154–E1164. Available from: https://journals.physiology.org/doi/abs/10.1152/ajpendo.00187.2013. 24022869 10.1152/ajpendo.00187.2013 PMC3840203

[24] Patil M, Belugin S, Mecklenburg J, et al. Prolactin Regulates Pain Responses via a Female-Selective Nociceptor-Specific Mechanism. iScience 2019 [cited 2023 Apr 16];20:449–465. Available from: https://pubmed.ncbi.nlm.nih.gov/31627131/31627131 10.1016/j.isci.2019.09.039 PMC6818331

[25] Paige C, Barba-Escobedo PA, Mecklenburg J, et al. Neuroendocrine mechanisms governing sex differences in hyperalgesic priming involve prolactin receptor sensory neuron signaling. J Neurosci. 2020 [[cited 2023 Apr 16]];40:7080–7090. Available from: https://pubmed.ncbi.nlm.nih.gov/32801151/32801151 10.1523/JNEUROSCI.1499-20.2020 PMC7480243

[26] Rochon J, Gondan M, Kieser M. To test or not to test: preliminary assessment of normality when comparing two independent samples. BMC Med Res Methodol . 2012 [cited 2023 Nov 20];12:1–11. doi: 10.1186/1471-2288-12-81. 22712852 PMC3444333

[27] Limón A, Pérez C, Vega R, et al. Ca2±activated K±current density is correlated with soma size in rat vestibular-afferent neurons in culture. J Neurophysiol. 2005 [[cited 2022 Jul 7]];94:3751–3761. Available from: https://pubmed.ncbi.nlm.nih.gov/16107534/16107534 10.1152/jn.00177.2005

[28] Ghovanloo M-R, Estacion M, Higerd-Rusli GP, et al. Inhibition of sodium conductance by cannabigerol contributes to a reduction of dorsal root ganglion neuron excitability. Br J Pharmacol . 2022 [cited 2022 Mar 17];179:4010–4030. doi: 10.1111/bph.15833. 35297036 PMC13012276

[29] Gawali VS, Todt H. Mechanism of inactivation in voltage-gated Na+ channels. Curr Top Membr. 2016;78:409–450. 27586291 10.1016/bs.ctm.2016.07.004

[30] Ghovanloo M-R, Aimar K, Ghadiry-Tavi R, et al. Physiology and pathophysiology of sodium channel inactivation. Curr Top Membr. 2016;78:479–509. 27586293 10.1016/bs.ctm.2016.04.001

[31] Fouda MA, Ghovanloo M-R, Ruben PC. Late sodium current: incomplete inactivation triggers seizures, myotonias, arrhythmias, and pain syndromes [Internet]. J Physiol. [cited 2022 Apr 18];600:2835–2851. Available from: https://pubmed.ncbi.nlm.nih.gov/35436004/35436004 10.1113/JP282768

[32] Hille B. Ion channels of excitable membranes. MA, USA: Sinauer; 2001.

[33] Waxman SG. Sodium channels, the electrogenisome and the electrogenistat: lessons and questions from the clinic. J Physiol. 2012 [[cited 2022 Nov 10]];590:2601–2612. Available from: https://pubmed.ncbi.nlm.nih.gov/22411010/. 22411010 10.1113/jphysiol.2012.228460 PMC3424719

[34] Ghovanloo MR, Effraim PR, Yuan JH, et al. Nav1.7 P610T mutation in two siblings with persistent ocular pain after corneal axon transection: impaired slow inactivation and hyperexcitable trigeminal neurons. J Neurophysiol. 2023 [[cited 2023 Feb 3]];129:609–618. Available from: https://pubmed.ncbi.nlm.nih.gov/36722722/36722722 10.1152/jn.00457.2022 PMC9988530

[35] Waxman SG, Zamponi GW. Regulating excitability of peripheral afferents: emerging ion channel targets. Nat Neurosci [Internet]. 2014 [cited 2023 Oct 23];17(2):153–163. Available from: https://pubmed.ncbi.nlm.nih.gov/24473263/24473263 10.1038/nn.3602

[36] Ahuja S, Mukund S, Deng L, et al. Structural basis of Nav1.7 inhibition by an isoform-selective small-molecule antagonist. Science. 2015;350:aac5464–aac5464. doi: 10.1126/science.aac5464. 26680203

[37] Alsaloum M, Higerd GP, Effraim PR, et al. Status of peripheral sodium channel blockers for non-addictive pain treatment. Nat Rev Neurol. 2020 [cited 2022 Dec 12];16(12):689–705. Available from: https://pubmed.ncbi.nlm.nih.gov/33110213/33110213 10.1038/s41582-020-00415-2

[38] Bankar G, Goodchild SJ, Howard S, et al. Selective NaV1.7 antagonists with long residence time show improved efficacy against inflammatory and neuropathic pain. Cell Rep. 2018 [cited 2018 Oct 3];24(12):3133–3145. Available from: http://www.ncbi.nlm.nih.gov/pubmed/30231997 30231997 10.1016/j.celrep.2018.08.063

